# Corn Stover for Food Applications: Approaches, Advances and Insights

**DOI:** 10.3390/molecules31010027

**Published:** 2025-12-22

**Authors:** Mariana Ochoa-Castaño, Nicolás Montoya-Escobar, Jorge Andrés Velásquez-Cock, Catalina Gómez-Hoyos

**Affiliations:** 1 Grupo de Investigaciones Agroindustriales, Universidad Pontificia Bolivariana, Circular 1 No. 70-01, Medellín 050031, Colombia; mariana.ochoac@upb.edu.co (M.O.-C.); nicolas.montoya@upb.edu.co (N.M.-E.); 2 Programa de Ingeniería en Nanotecnología, Universidad Pontificia Bolivariana, Circular 1 No. 70-01, Medellín 050031, Colombia; jorgeandres.velasquez@upb.edu.co

**Keywords:** corn, corn stover, corn by-products, food applications, biomolecules, circular economy

## Abstract

Corn processing generates substantial volumes of agricultural by-products, collectively referred to as corn stover, comprising husks, cobs, stalks, leaves, and silks. Although rich in bioactive compounds, these by-products are still predominantly destined for low-value uses such as landfilling and open-field burning. They contain valuable biomolecules such as lignocellulosic fibers, starch, pectin, proteins, and polyphenols, all of which hold significant potential for applications in agricultural and food industries. These compounds offer opportunities as sustainable alternatives to conventional ingredients and as novel functional additives. However, utilization of corn stover remains focused on biofuel production, limiting the development of applications in broader, high-value fields such as functional food ingredients. This review aims to highlight the opportunities that corn stover presents for developing solutions for food production, which is becoming increasingly important as the global population continues to grow and food demand rises, particularly in regions where access to sufficient and nutritious food remains limited. It also considers the challenges to be solved in order to incorporate corn stover in circular economies, like the impact of pesticide presence on derived products and gaps of emerging strategies for scaling up production in alignment with circular economy goals and the high-value utilization of corn stover.

## 1. Introduction

Corn, or maize (*Zea mays* L.), is a cereal crop domesticated in Mexico about 9000 years ago. It ranks among the most important crops worldwide, along with wheat and rice, serving as a staple food source [1]. Globally, nearly 208 million ha are used for maize production, with growing regions in sub-Saharan Africa, Asia, and Latin America [2]. While it is cultivated primarily in the Americas and Asia, each with over one-third of the total areas of cultivation [3], its output is dominated by the United States and China, yielding approximately 361 and 259 million t per year, respectively. Maize production primarily focuses on its kernels, which comprise 30–52 wt.% of the total plant biomass [4] and are used for human consumption and as a source for biodiesel and bioethanol [5,6]. A major industrial product derived from corn is starch, obtained through wet and dry milling. These processes generate several well-known by-products, hereinafter referred to as processing corn by-products, such as corn oil, corn gluten meal, and corn steep liquor, among others [7]. Extensive research has been conducted on the use of these by-products [8,9,10], and significant advances have been made in extracting plant-based proteins from sources like corn gluten meal [11].

**Figure 1:**
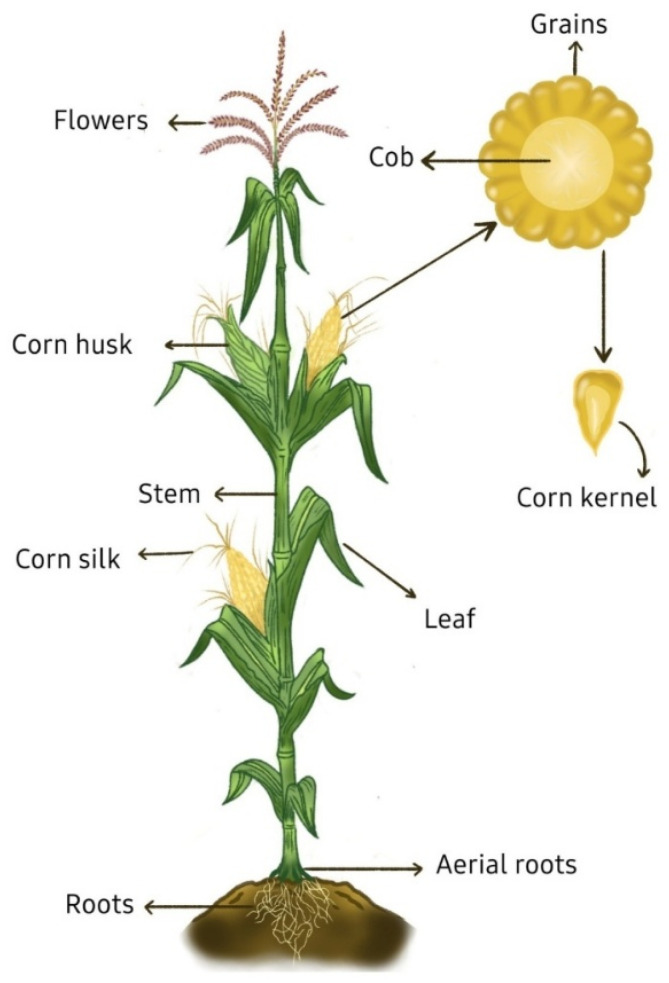
Schematic representation of the corn plant (*Zea mays* L.), showing its main anatomical structures.

Nonetheless, while most research has primarily concentrated on processing by-products [8,10,12,13], they are not the only residues generated by this industry. A great portion of the agricultural by-products of corn remain unutilized, especially corn stover, comprised by stalks, leaves, husks, cobs, and silks. Figure 1 shows the part of the corn plant from where these by-products come. The proportion of corn stover relative to the total plant biomass varies according to different studies, with values ranging from 0.65 to 0.86 on a dry weight basis, depending on factors such as corn variety, harvesting practices, and growing conditions [1]. Despite these variations, the quantity of corn stover generated is enough to justify further research on its valorization for economic and environmental benefits; for example, in 2023, corn production reached 1.2 billion t which corresponds to approximately 900 million t of stover [2]. In fact, corn waste has been identified as a major contributor to greenhouse gas emissions, both from landfilling and biomass burning, making the development of utilization strategies an urgent matter [14].

Beyond their environmental implications, corn stover represents a source of bioactive compounds, including polysaccharides [15], proteins [16], and phenolic compounds [17], which can be extracted and utilized for industrial applications. Polysaccharides, such as cellulose and hemicellulose, are the primary components in corn stover, along with smaller amounts of lignin, starches, and xylans [7,9]. It is worth mentioning that the distribution of these compounds changes among the different stover components [18,19], which is important, as they often determine which stover component is selected for the extraction of a specific biomolecule. In the case of proteins, they have been largely extracted from corn silks and corn cobs [20], while phenolic compounds, which are studied primarily for their antioxidant activity, are mainly isolated from corn silks [21].

Several works have studied these biomolecules and emphasized corn stover potential in the development of food additives [8,22]. Thus, corn stover presents an opportunity for the development of solutions for food production [13], which becomes increasingly urgent as global population growth continues, and food demand rises, particularly in regions where access to sufficient and nutritious food remains limited [23]. However, utilization of corn stover remains largely concentrated on biofuel production [24], limiting the development of applications in fields such as functional food ingredients. 

This review, therefore, aims to summarize advances in the characterization and valorization of corn stover for food applications, while also addressing the current lack of studies that integrate its potential to tackle the challenges of developing new food ingredients. Thus, this paper performed an analysis of over 100 articles, including peer-reviewed papers, grey literature, and authoritative reports, with a focus on the valorization of corn stover for food applications (specifically polysaccharides, proteins, and phenolic compounds derived from corn stover). While the primary sources were published between 2003 and 2025, earlier studies were included when they were deemed essential to certain research areas. The selected literature was assessed for its relevance to food security, sustainability, and agricultural practices. The review provides a comprehensive foundation for future research and policy by highlighting a gap in current literature, which tends to focus on biofuels rather than the use of corn stover for food. 

## 2. By-Products Across Corn Production Chain and Its Applications

**Figure 2:**
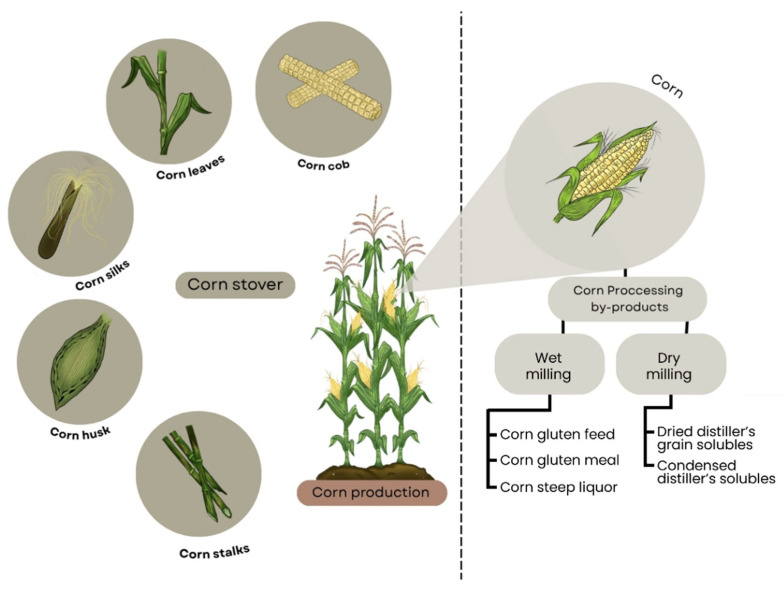
Scheme for corn (*Zea mays* L.) showing both plant residues (corn stover) and industrial by-products from milling processes (corn processing by-products). Chart adapted from Ruan et al. [7].

Corn production chain comprises different stages, starting with farming, where corn is planted, grown, and harvested, then moves to processing to create various products, such as cornmeal or ethanol [25]. After harvesting, several plant by-products are obtained, such as corn husk, cob, stalk, silk and leaves, which comprise the corn stover [1]. The grain is processed for its packaging and distribution as canned food or processed to manufacture different products. Figure 2 presents a scheme that includes corn stover and corn processing by-products. 

The most common techniques used to process the grains are wet milling and dry milling [10]. In dry milling, corn grains are degermed and milled to obtain corn flour and flakes [26], while wet milling steeps the grains in water and sulfur dioxide to facilitate the subsequent milling, thereby enhancing the separation and recovery of components such as starch. This same process also enables the recovery of corn oil from the germ [27]. As observed in Figure 2, these operations produce different by-products, named in the present work as “corn processing by-products”, and have been widely studied as evidenced by the reviews from [7,12,25].

Valorization of corn stover would align with successful strategies already reported for other agricultural by-products. For example, several countries have seen the exploitation of cocoa by-products as an opportunity with both economic and productive advantages; in this context, scientific research has evaluated the incorporation of cocoa by-products into the formulation of low-calorie, high-fiber foods such as chocolate cookies, cakes, and dietary supplements [28,29,30,31]. Similarly, commercial products like cocoa shell tea [32] and cocoa shell flour [33] have been developed. These efforts have driven the commercialization of cocoa by-products and products derived from them, resulting in the export of 279,239 t of cocoa by-products by 2024 [34]. 

As with cocoa by-products, biomolecules present in corn stover highlight its potential as a raw material for functional and nutritional applications. Preliminary studies show interesting results for the development of functional ingredients [35,36], nutritionally enhanced products [35,37], and novel food additives [37,38,39]. Agro-industrial uses have also emerged, including applications in soil enrichment, sustainable fertilization, and plant protection [40]. An overview of the relevant developments could help to highlight the most promising applications and drive the progress of this field.

### Representative Applications of Corn Stover

**Figure 3:**
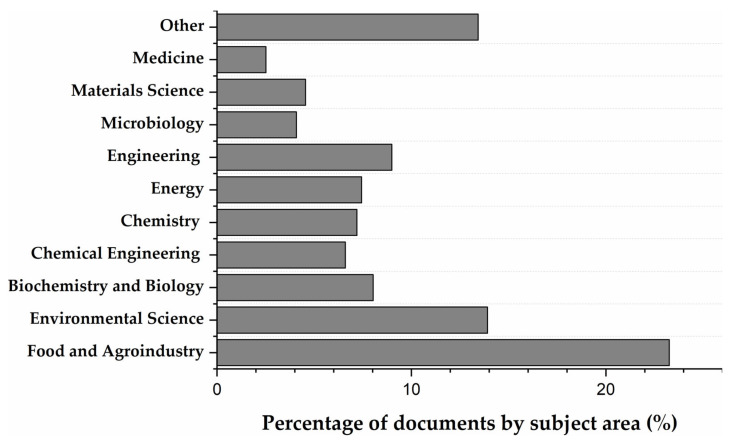
Research by subject area. Data obtained from Scopus using the equation: (TITLE-ABS-KEY ((corn OR maize) AND by-products) AND TITLE-ABS-KEY (cob OR husk OR leaves OR silk) AND ALL (food OR application)) AND PUBYEAR > 2003.

Beyond these sectors just mentioned, other industries have utilized corn stover in areas such as pharmaceuticals, cosmetics, and material synthesis. Figure 3 shows the percentage of research done by area of application. The analysis reveals that Food and Agroindustry is the most prominent area, accounting for over 22% of the research, followed by Environmental Science (about 15%). Areas such as Biochemistry and Biology (around 9%), Energy (7%) and Materials Science (5%), show moderate representation, which suggests ongoing exploration of the individual components of corn stover in bioenergy production, material development (e.g., bioplastics, composites) [41,42,43,44].

In the agroindustry sector, corn stover has contributed to sustainable agricultural practices, as improved soil health, and enhanced crop productivity [45,46]. Ullah and coworkers conducted a two-year factorial experiment to evaluate the combined effect of stover return and phosphate fertilization on sweet corn yield [47]. By applying different phosphate fertilizers (DAP, SSP, and NP) along with corn stover, the study revealed that incorporating agricultural by-products into soil management strategies could reduce the need for synthetic fertilizers. It also demonstrated that co-application of 5 t per ha of corn stover with 90 kg/ha of diammonium phosphate (DAP) enhanced sweet corn yield. The findings suggest that such organic amendments may improve soil fertility and phosphorus use efficiency, potentially enabling a gradual reduction in reliance on conventional fertilizers [47].

In addition to soil enhancement, corn stover also plays a role in preventing plant diseases. P. Yang et al. [48] patented a microbial consortium designed to prevent and treat fungal diseases in plant leaves. It integrates part of corn stover, such as corn silk, with *Bacillus siamensis*, biochemical potassium fulvate, polyglutamic acid, and potassium phosphite. The synergistic effect of these components enhanced plant stress resistance, increased the defensive enzyme activity, and improved overall disease resistance. Additionally, it reduced toxic substances, such as malonaldehyde in plant cells, promoted nutrient accumulation in leaves, and increased chlorophyll content, ultimately leading to healthier crops [48].

Besides the applications mentioned above, Table 1 summarizes other studies conducted with corn stover, among which stands out its use in the food and energy sector. In the food sector, various biomolecules derived from corn stover have been isolated and applied as functional ingredients. For instance, xylooligosaccharides (XOS) extracted from corn cobs have demonstrated the ability to stimulate the growth of probiotic organisms [37]. Similarly, the utilization of corn silk to produce extracts rich in phenolic compounds with antioxidant and inhibitory activity has been explored [49]; these extracts have potential for food or cosmeceutical applications [36,50]. Moreover, in the energy sector, components of corn stover have been utilized in the fabrication of porous electrodes for capacitors [51] and as precursors of carbonaceous materials, offering a sustainable alternative to graphite in the anodes of lithium-ion batteries [52].

In the livestock sector, corn stover components are commonly dried and milled into fine powders for incorporation into animal feed and silage formulations that have been evaluated in diverse applications, such as mealworm feed, broiler feed, dairy rations, and novel dietary supplements for cattle and sheep [53,54,55]. In the field of materials engineering, corn stover has been employed in the development of sound-absorbing materials, with performance evaluated according to the ASTM E1050 standard [41]. Fibers extracted from corn stover have also been fractioned into cellulose for subsequent use in paper sheet production or for the enhancement of mechanical properties [42,43]. In a similar approach, corn cob ash was incorporated into cement milling processes, partially replacing Ordinary Portland Cement clinkers at substitution levels ranging from 2 to 25 wt.% [44]. Furthermore, dried corn cobs have been utilized for the synthesis of MgO–biochar nanocomposites via magnesium activation under a nitrogen atmosphere at 400 °C and 500 °C, which have shown effectiveness in removing NH_4_^+^ and PO_4_^3−^ from water through chemisorption and physisorption mechanisms [56].

Since most applications of corn stover reported in the literature are focused on the food sector, these will be examined in greater detail in a subsequent section. This focus on food is relevant for corn, as it is principally cultivated for the use of its kernels in this field. In addition, extending the valorization of corn stover to food applications aligns naturally with this primary purpose and supports an integrated use of the crop, promoting responsible consumption and production practices in accordance with Sustainable Development Goal (SDG) 12.

**Table 1:** Applications of corn stover: industrial areas, products, processing methods and potential uses.

Area	Product	Corn Treatment/Production Method	Used/Potential Uses	Reference
Agroindustry	Soil conditioner	Corn stover from a previous crop is chopped, mixed, and incorporated into the field with the help of a rotavator. The corn stover return has been mixed with phosphate fertilizer	Use for improvement of soil properties	[40]
Agroindustry	Animal Feed	Corn cobs, stalks and leaves are dried and milled to obtain a fine powder which is added to the animal feed and subsequent silage.	Feed for mealworm; dairy feed; novel feed for cattle and sheep; fermented broiler feed; geese feed for meat production	[53,54,57,58,59]
Biotechnology	Solid support in solid state fermentation (SSF)	Corn cob is dried in oven for 48 h and ground to 40-mesh (400 µm) or less. Then, it is used as a carbon source in fermentation	Nutrient in solid state fermentation processes to produce antimicrobials and antioxidant agents	[60,61]
Biotechnology	Lignocellulosic fiber	Looking to expose the hydroxyl groups, dried corn cob is treated with alkali (2 mol NaOH) in a stirred suspension for 24 h. The pretreated support is activated with glyoxyl groups, glutaraldehyde and IDA-glyoxy. Then, the immobilization of trypsin is performed in the presence of 3 mmol/L of benzamidine (a competitive inhibitor of trypsin) to prevent autolysis.	Enzyme immobilization	[62]
Cosmetic	Facial cream	Polyphenols from corn silk are extracted in ethanol and ethyl acetate and incorporated into a facial cream product	Anti-Tyrosinase, and Anti-Skin Pathogenic Bacterial Activities	[49]
Energy	Anodes	Corn stalk is hydrolyzed under basic or acidic conditions, and then activated chemically or by further heating, under controlled atmosphere.	Anodes for energy storage in lithium-ion batteries.	[52,63,64,65]
Energy	Porous Electrodes	Corn cobs, leaves, stalks, husk or silk, are submitted to hydrothermal treatment with acids or bases and then activated using temperature or KOH	Electrodes for supercapacitors	[51,63,66]
Energy	Substrate for dark fermentation	Corn cobs are delignified and/or saccharified, to obtain fermentable compounds.	Fermentative hydrogen production	[67]
Energy	Ethanol	Ethanol production is achieved from corn cobs following a combined thermochemical and fermentative biorefinery approach, with yields comparable to results in conventional pretreatments and fermentation processes.	Biofuel production	[8,24]
Energy	Gasoline	Corn leaves and stalks are processed by grinding, adding auxiliary materials (leaven and water) and fermenting	Preparation of biological gasoline	[68]
Food	Xylooligosaccharides (XOS) extract	XOS are extracted from corn cob using autohydrolysis, acid hydrolysis and enzymatic hydrolysis. Then, its separation and purification are made using nanofiltration, ethanol elution, gel chromatography, or ion exchanging.	Xylooligosaccharides with capability to stimulate the growth of probiotic organisms	[37,39]
Food	Xylitol	Corn leaves, corn cob and corn husk are grounded and later mixed with acid and refluxed. The obtained sugars are reduced slowly using NaBH_4_ or other reducing agents.	Sugar substitute with specific health claims	[38]
Food	Bioactive packaging	The polyphenols from corn cob are extracted and incorporated into an alginate film solution which can be prepared by dissolving 1% (*w*/*v*) sodium alginate and 0.5% (*w*/*v*) CaCl_2_ into deionized water using glycerol as a plasticizer.	Use of polyphenols in edible films to impart antimicrobial activity	[69]
Food	Fiber	Corn husks are washed, dried, and milled into fine powder. The powder undergoes an alkaline treatment, bleaching and is centrifuged to obtain microcrystalline cellulose which is freeze-dried and stored. Microcrystalline cellulose is employed to balance the covalent cross-linking reaction between proteins in a soy protein film.	Packaging reinforcement	[70]
Food	Functional ingredient	Cellulose is isolated from corn stover through alkaline and acid treatments, and polyphenols are extracted using ultrasound-assisted extraction and exploring different solvents	Additive in food matrices to enhance its nutraceutical properties or use the fiber as stabilizer.	[35,36]
Food	Dietary Fiber	Corn silk is dried and grinded to obtain a fine powder for the development of biscuits	Development of biscuits that have good sensory quality, low-fat, sugar-free type, long satiety time and high nutritional value	[71]
Food	Straws	Corn husk is milled and mixed with xanthan gum, carnauba wax and stearic acid. A binder ingredient can be added resulting in a smooth and hard, durable and biodegradable material, which is extruded to form straws.	Production of biodegradable materials for their use in the food industry	[72]
Food/Health/Cosmetic	Polyphenols extract	Extraction from corn silk, corn husk or purple corn cob using different methods: maceration, heating, ultrasound-assisted extraction, microwave assisted extraction.	Antioxidant activities; cosmeceutical potential including tyrosinase inhibition and anti-aging activity; bioactive compounds to incorporate in food matrices.	[36,50,73,74]
Materials	Sound absorbing material	The corn husk is repeatedly washed with distilled water to remove dust, dirt and impurities, then it is air dried and cut to a specified size to build single and multi-layer corn husk systems with different back cavity. The acoustic absorption is analyzed based on the method of ASTM E 1050 [75].	Corn husk for noise reduction applications	[41]
Materials	Fiber	Corn leaves and stalks are fractionated into cellulose fibers, sugars, and lignin by a treatment using aqueous formic acid. A paper sheet is prepared using the obtained cellulose fibers	Reinforcement in paper sheet for enhanced mechanical properties	[42]
Materials	Furfural	Corn cob is hydrolyzed and a synergistic catalytic mechanism for transforming xylose-rich corncob-hydrolysate into furfural were proposed using SO_4_^2−^/SnO_2_-FFS as a chemocatalyst in DESMLA–water containing ZnCl_2_.	Precursors for the fabrication of chemical substances	[76]
Materials	Precursor chemicals	Levulinic acid, formic acid, and furfural, are synthesized from corn husk by hydrothermal conversion using an acidic catalyst and a statistical Box–Behnken method.	Precursors used in pharmaceutical and cosmetic industry	[77]
Materials	Fiber	Dried corn cobs are ground and burnt in open air, using a local blacksmith furnace that uses charcoal as fuel until corn cob turned to ashes. The corn cob ashes are used in cement milling, replacing different percentages by weight of Ordinary Portland Cement clinkers.	Corn cob ashes for use as a pozzolan in blended cement	[43,44]
Materials	Xylan extract	Corn cob is dried, and ground until it turns into flour. Xylan extraction is carried by adding NaOH (1.8 M) to corn flour combined with ultrasonic waves. The suspension is then centrifuged to separate the soluble portion, containing xylan.	Precursors for nanoparticle synthesis, such as, antifungal silver nanoparticles	[78]
Materials	Lactic acid	The corn cob was preprocessed with an acid treatment. The resulting fibrous residues and acidolysis liquid were subjected to enzymolysis. The treated mixture was then used as a fermentation substrate, where microbial fermentation was carried out to produce lactic acid.	Food ingredient	[79]
Water treatment	MgO-biochar	Dried corn cob is immersed into MgCl_2_ solution (3.3 M) which is mixed continuously by a magnetic stirrer for 2 h. The mixture is then dried at 80 °C. The dry mixture is used to prepare MgO-biochar nanocomposite using a 3-zones quartz tube furnace	Removal of NH_4_^+^ and PO_4_^3−^ from water	[56]
Effluents treatment	Corn cob powder	Corn cob is dried at 70 °C in an oven for 24 h. The dried corn cobs are crushed and then sieved by passing it through mesh sizes of 60–200. Finally, 0.075 mm size particles of corncob are selected as an adsorption media.	Treatment of textile wastewater generated from the dying, printing, and finishing processes	[80]

## 3. Corn Chemical Composition

Proximate analysis provides a quick overview of the basic chemical composition of raw materials, making it particularly valuable in fields such as food and agroindustry. In the context of corn stover, a proximate analysis of the main residues obtained directly from the corn plant allows to quantify parameters such as moisture, volatiles, fixed carbon, ash, and protein content, key parameters for their utilization [81]. 

According to experimental data, reported in Table 2, the moisture content of these residues ranges from ~4 to ~9 wt.% in stalks [19], while silk exhibits a higher moisture level (~84 wt.%) [82]. Volatile matter varies from ~73 to ~88 wt.%, depending on the analyzed by-product [83]. Fixed carbon content also fluctuates, with values between ~8 and ~28 wt.%, which may influence their use as biomass for energy generation [84]. In the case of ash content, studies have shown that its levels range between 2 and 18 wt.%. For instance, husks contain between ~8 and ~18 wt.% ash, whereas cobs have a lower content of ~2 wt.% [85]. As for the protein fraction, values range from ~2 wt.% in stalks to ~18 wt.% in silk, indicating potential applications in food and nutraceutical industries due to its ability to facilitate processes such as solubilization, emulsification, foaming, gelation or dough formation [86] and for its contribution to the protein content of any food preparation [87]. 

The compositional difference in the corn stover components suggests that each one can be utilized in a differentiated manner, depending on its physicochemical properties and its macronutrient and mineral content. Table 2 presents the details of the proximal analysis of each component. It is important to emphasize that variations in proximate composition are also influenced by the corn variety being analyzed [88]. The considerable genetic diversity among corn cultivars results in distinct nutritional profiles, which can, in turn, determine the most appropriate applications for each variety in food, feed, or industrial contexts [89]. Nonetheless, research efforts have largely focused on the commercial variety *Zea mays* L., with limited exploration of native cultivars and their potential uses.

**Table 2:** Proximal analysis of different parts of corn stover.

Corn by-Product	Moisture	Volatile Matter	Fixed Carbon	Ash	Protein	Reference
	(wt.%) ^1^					
Corn husk	~7–9	~75	~14	~8–18	~12	[19]
Corn leaves	~6–7	~75	~11	~15–18	~12–15	[18,83]
Corn stalk	~4–10	~73–85	~8–21	~2–6	~2	[19,84,90]
Corn cob	~6–7	~74–88	~10–18	~2	~3	[19,85]
Corn silk	~84	Not reported	~28 (Carb.^2^) ~48 (F.D.^3^)	~4–5	~9–18	[86]

^1^ Values were rounded up to the nearest full percents. ^2^ Carb. refers to carbohydrates; ^3^ F.D. refers to dietary fiber.

Besides the components characterized through proximate analysis, corn stover is rich in a variety of biomolecules such as lignocellulosic fibers, polysaccharides, polyphenols, and proteins, which stand out due to their high content and functional properties [91,92,93]. These compounds constitute the key contributors facilitating the valorization of corn stover across the multiple sectors shown in Table 1, including bioenergy [6], food technology [10], pharmaceuticals [94], and materials science [44]. The following section delves into the characteristics and potential applications of these biomolecules, highlighting their importance in the comprehensive utilization of corn stover.

### 3.1. Structural Biopolymers in Corn Stover

Lignocellulosic fibers present in corn stover are composed of around 35–50 wt.% cellulose, 20–35 wt.% hemicelluloses and 10–25 wt.% lignin [95]. Cellulose is a β-glucan linear polymer composed of glucose molecules linked by β-(1,4) bonds [96]. Its highly ordered structure, reinforced by hydrogen bonding between adjacent chains, contributes to its stability and mechanical strength [97]. Hemicellulose is an amorphous polysaccharide with a lower molecular weight than cellulose [98]. It is composed of a mixture of pentoses, making it more flexible and less crystalline [99]. In contrast, lignin is characterized by its aromatic structure formed through C–C and C–O–C bonds that link phenylpropane units [99]. Functionally, lignin acts as a binding agent between cellulose and hemicellulose, providing rigidity and structural support to plant fibers [95].

Table 3 presents the variation in these lignocellulosic components across corn stover. For cellulose, its content ranges from ~18 to ~38 wt.%, with the highest levels found in corn husk (~29–38 wt.%), and the lowest in corn leaves (~19 wt.%) and corn cobs (~18–30 wt.%). In the case of hemicellulose, it shows the widest variation, from ~33 to ~45 wt.%, with corn cob and corn husk containing the highest amounts (~45 and ~44 wt.% respectively). Finally, lignin content varies from ~7 to ~22 wt.% and is most abundant in corn stalks (up to ~20 wt.%) and corn cobs (~22 wt.%). The high content of these biomolecules contributes to the overall lignocellulosic richness of corn stover, which has been highlighted by Hidayatullah et al. [15] as a valuable attribute for their application in the fiber and composite industries. In fact, H. Wang et al. [100] reported that most fibers in corn cobs are insoluble and form a three-dimensional network capable of effectively trapping both water and oil, making it highly absorbent and supporting the increasing interest for its use in different industries.

Hemicelluloses, cellulose and lignin constitute insoluble fibers that have been consumed for centuries and are recognized for their health benefits [102]. For this reason, husks and cobs are gaining interest for their potential as rich sources of insoluble fiber in food systems [103,104]. In addition, these materials contribute significantly to the texture, cohesiveness, and moisture retention of gluten-free and extruded products [105,106]. Their integration into food matrices has been shown to enhance bulk and reduce syneresis, especially in high-fiber formulations like snacks, batters, and plant-based gels [107]. Furthermore, optimized processing methods, such as grinding or ultrasonic-microwave treatment, can tailor their surface activity and improve dispersibility in composite flours [108]. Thus, the functional and nutritional roles of insoluble fiber position it as a promising candidate for texture enhancement and health-oriented applications in food formulations [104]. 

Among lignocellulosic fibers, cellulose is the most widely explored component in corn stover, especially when processed into nanostructures [15]. Cellulose nanofibrils are nano-scaled fibrils derived from cellulose and obtained through mechanical treatment of this polysaccharide [109]; they have high surface area (150–250 m^2^/g) [110], biodegradability, and tunable surface chemistry [111], which combined with their lightweight and renewable nature, make them suitable for food applications. In fact, since 1980, Turbak and coworkers outlined its potential applications as a food additive [112]. Within this scope, nanocellulose fibers act as rheology modifiers [113], emulsion stabilizers [109], and moisture retainers [114]. Cellulose nanofibrils can be extracted from corn cob [15], husk [70], stalk [115], leaves [116], and silk [16]. In this sense, corn stover is a promising precursor of this biomolecule and a sustainable alternative to wood-derived sources [117], facilitating its extraction in regions where the supply of fast-growing wood species is limited.

### 3.2. Non-Structural Biopolymers in Corn Stover

Beyond the polysaccharide components of lignocellulosic fibers (such as cellulose and hemicellulose) which provide structural support in corn stover, other non-structural polysaccharides also play significant roles and offer valuable applications. Starch is primarily found in residual amounts in corn cobs [118], while pectins are more abundant in softer tissues such as corn silks and leaves [119]; lastly, xylans (particularly arabinoxylans) are present in different corn fractions, including husks and cobs [38]. These polysaccharides, often overlooked in favor of cellulose and hemicellulose, offer valuable opportunities for biotechnological and food-related applications.

Chemically, starch is a polymer formed by amylopectin and amylose, which are glucose-based polymers linked through glycosidic bonds [120]. The proportion between amylose and amylopectin will determine different properties of the resulting starch; while amylose is water insoluble and contributes towards increasing the gelatinization capacity of starch, amylopectin readily dissolves in water and is used to enhance its adhesive properties [120].These properties provide the functionality of starch in the food industry, where it is used as a gelling, thickening and/or stabilizing agent, as well as being used as a binder, sweetener and emulsifier [118]. Among the components of corn stover, corn cobs have been utilized as a source of starch, with reported contents ranging from approximately ~10 to ~13 wt.% [100]; this makes them a sustainable and economical raw material for producing eco-friendly edible films [121].

Pectins are complex heteropolysaccharides primarily composed of α-(1→4)-linked D-galacturonic acid units [122]. Overall, pectins are primarily found in the primary cell walls and intercellular regions of plants, but in corn, they are predominantly located in softer tissues such as silks, leaves, and husks [123]. These polysaccharides contribute to the structural integrity of the plant, by forming gel-like networks that provide mechanical strength and flexibility [123]. Pectins have been studied due to their potential applications in various industries, particularly in the food sector, where they are used as gelling agents, stabilizers and thickeners in the production of jams, jellies and similar food products, but they also exhibit potential as prebiotics, promoting the growth of beneficial gut bacteria and offering health benefits [124]. 

For example, Higuera-Coelho et al. [119] extracted pectin from corn husk, utilizing a combination of high-power ultrasound and alkaline and enzymatic treatments. With this method, they increased the yield and solubility of pectin, compared to conventional extraction methods, while obtaining pectins with high uronic acid content (67 wt.%) and low levels of methylation and acetylation [119]. The obtained pectins were characterized through molecular weight estimation, atomic force microscopy and rheological assessments, demonstrating that the treated pectin exhibited improved water solubility and smaller hydrodynamic sizes. Notably, this work reported that interactions between pectin and calcium or iron were of interest their potential for nutrient delivery applications: while calcium induced irreversible gel formation, iron formed reversible gels, both of which could be used for distinct functional uses [119]. With this, corn husk pectins are highlighted as sustainable biopolymers for food and pharmaceutical industries, where they could function as texture modifiers and facilitate nutrient delivery. 

Although hemicelluloses were addressed in the context of lignocellulosic fibers, they are reiterated here with particular emphasis on xylan, a specific polysaccharide which forms hemicellulose and is prevalent in corn stover [125]. Xylan is a branched polymer consisting of β-1,4-linked D-xylopyranose (xylose) units, with various side chains such as arabinose, glucuronic acids, and other residues [126]. This biopolymer can be derived from various agro-industrial by-products, particularly those rich in lignocellulosic fibers, such as corn cobs, husks and stalks [127]. Among the corn stover, corn cobs have the highest xylan content, accounting for 40 wt.% [126]. While xylan itself has been widely studied, increasing attention has also been directed toward its hydrolysis products, such as xylose and xylooligosaccharides (XOS), due to their potential applications as soluble sugars and prebiotic ingredients in the food industry. Both xylose and XOS can be obtained from xylan through enzymatic or chemical treatment. Xylose has been studied as a precursor for xylitol, a sugar alcohol widely used as a low-calorie sweetener with dental health benefits [128]. 

Concerning xylan isolation, different segments of corn stover have been evaluated as possible sources for the extraction of both xylose and XOS. For example, Samanta et al. [39] outlined an efficient bioconversion process for corn cob valorization through alkaline extraction of xylan, achieving over 80% yield with 12% NaOH and steam. Subsequent hydrolysis with dilute sulfuric acid produced xylooligosaccharides (XOS), primarily xylobiose and xylotriose. In vitro assays confirmed the prebiotic potential of XOS, particularly in stimulating the bacteria *Enterococcus faecium*, highlighting its relevance as a functional ingredient in food formulations aimed at gut health improvement. Similarly, Egüés et al. [129] studied the potential of corn stalk-derived hemicelluloses as functional ingredients in food systems. Alkaline extraction, especially when preceded by dewaxing and delignification, enabled recovery of up to 54% of hemicelluloses with minimal lignin impurities, yielding material with favorable properties for use as dietary fibers, stabilizers, or texture modifiers [129]. 

Qian et al. [130] also studied the effects of enzymatic hydrolysis with xylanase to produce XOS from corn stalks. Similar treatments were proposed by authors such as Samanta et al. [91] and Chapla et al. [131], although using two different sources, corn husks and corn cobs, respectively. Overall, most researchers focus on the extraction of xylose and XOS from corn stover through enzymatic treatments, but further research should be directed to propose alternative methods for the isolation of these polysaccharides, combining these treatments with other physical or chemical methods, in order to standardize treatments that have higher yield, purity and lower production costs [132].

### 3.3. Polyphenols in Corn Stover

Polyphenols or phenolic compounds are found as secondary metabolites in plants [133], which use these compounds for lignin and pigment biosynthesis, protection against invading organisms, growth, reproduction and other key functions [134]. They are also important components of human diet due to their wide range of physiological properties, such as, antihypertensive, antibacterial, antiviral, antiobesity, anti-inflammatory, vaso-dilating, UV-protecting, among others [135,136,137,138,139].

Corn stover has been consistently reported to contain these biomolecules [17]. Throughout the different corn varieties, purple corn stover exhibits a greater total phenolic content (TPC) than its white or yellow counterparts, a difference likely associated with the higher concentration of anthocyanin [74,140]. Within a same variety, cob and husk generally display comparable TPC ranges, while corn silk presents the greatest values of TPC, ranging from ~35–38 mg GAE/g dw in white or yellow corn to ~52–55 mg GAE/g dw in purple corn (Table 4).

Cai et al. [141] assessed the antioxidant profile of husk from 15 specimens of yellow corn and 8 specimens of waxy corn. Ferulic acid was identified as the major non-anthocyanin polyphenolic compound for both types of corn, with 118.85 µg/g as the highest content in yellow corn and 1101.90 µg/g for waxy corn. Isofraxidin was also observed in most corn cultivars. The highest content of isofraxidin among yellow corn was 70.52 µg/g, and 75.58 µg/g among waxy corn. Waxy corn husk extract was also observed containing rutin (0.80 µg/g), quercetin (14.48 µg/g), and dihydroluteolin (170.48 µg/g). For other common flavonoids found in corn, naringenin, hesperetin, coumarin, and coumaric were also identified in lower proportions and fewer varieties. In another study, Dong et al. [142] recognized 20 phenolic compounds, encompassing 6 flavonols, 1 dihydroflavonoids, 2 biflavonoids, 2 flavanons, 4 cinnamic acids, 3 anthocyanin and 2 coumarins. 

**Table 4:** Polyphenol content measured by Folin-Ciocalteau method in corn stover.

Corn Type	Corn Stover Component	$\mathrm{TPC}\;(mg_{GAE}/{g_{dw}}^{1})$	Reference
*White/Yellow* ^2^	Cob	~2–3	[100,142]
	Husk	~2–3	[141,142]
	Silk	~35–38	[143]
*Purple corn*	Cob	~2–29	[21,144]
	Husk	~23–27	[144]
	Silk	~52–55	[143]

^1^ Values were rounded up to the nearest full percents. ^2^ Stover of white and yellow corn have similar phenolic content, but purple corn stover differ from them.

Similarly, Lau et al. [145] studied the phytochemical composition of sweet corn cob from a mixed variety. The authors identified ferulic and *p*-coumaric acid as the major components, similar to the findings of Cai and coworkers [141] in corn husk. The total amount of ferulic and *p*-coumaric acid present in the corn cob free phenolics fraction was 100 µg/g and 150 µg/g, respectively. These results align with previous investigations indicating that the ferulic acid and *p*-coumaric acid content in yellow corn ranges from 6 to 1800 µg/g [146,147]. 

Beyond corn husk and cob, corn silk has been the most investigated component of corn stover for extracting polyphenols. A study by Lapčík et al. [148] examined the antioxidant properties of corn silk extracts and identified several key compounds, including ferulic acid, chlorogenic acid, epicatechin, kaempferol, rutin, and vanillic acid. The concentration of these compounds depends largely on the extraction temperature. Among them, chlorogenic acid (97.58–183.29 μg/g), epicatechin (8.55–31.23 μg/g), ellagic acid (17.34–122.69 μg/g), vanillic acid (3.02–88.86 μg/g), rutin (3.32–10.79 μg/g), and ferulic acid (1.27–3.48 μg/g) tend to be the most abundant in corn silk. However, the levels of these polyphenols can vary significantly based on several factors, such as the plant maturity stage, whether the corn silk has been pollinated, and the activity of enzymes like polyphenol oxidase [149,150]. Additionally, differences in corn variety, cultivation conditions, and soil quality can also influence the overall polyphenol content [151].

While the content of polyphenols present in corn silk is well-established, the polyphenolic composition of corn leaves and stems has been less explored. Vazquez et al. [152] reported a total phenolic content of free phenolic extract of 2.6 and 4.8 mg GAE/g dw for leaves and stems, respectively. However, these parts of the plant are predominantly used for biodiesel production, thus research efforts have concentrated in their transformation for energy applications, reducing the information on these bioactive compounds [8].

As mentioned previously, the variety of corn has also an important influence on the polyphenol content. Ratha et al. [140] assessed the phytochemical composition of purple corn, investigating cob, silk, and husk. Results indicated that purple corn silk extract showed the highest total phenolic content (650 mg GAE/g extract) and antioxidant activities (Half-maximal Inhibitory Concentration IC_50_ = 17.24 µg/mL for DPPH assay). Phenolic compounds present in the extracts were composed of gallic acid, protocatechuic acid, *p*-hydroxybenzoic acid, chlorogenic acid, vanillic acid, *p*-coumaric acid and ferulic acid. Protocatechuic acid, *p*-hydroxybenzoic acid and vanillic acid were the major phenolic acids observed in all the purple corn extracts. These results supported a previous work of Chutikarn Kapcum et al. [153] who found protocatechuic to be the major phenolic compound in the pericarp, silk, and cob of purple corn. 

Ratha et al. [140] also reported the presence of anthocyanins in purple corn stover. The total anthocyanin content (TAC) ranged from 10.0 to 18.3 mg per 100 g of extract. The highest TAC value was recorded in the cob extract (18.39 mg/100 g), followed by husk extract (13.20 mg/100 g) and silk extract (10.18 mg/100 g). Delphinidin, one of the major anthocyanin substances responsible for the deep blue-to-purple color in plants and with strong antioxidant, anti-inflammatory, anticancer, and cardiometabolic benefits, among others [154], presented contents in the range of 6.0–11.0 mg/g, where the cob extract exhibited a significantly higher delphinidin content than the other individual extracts [155]. 

Thus, corn stover contains a diverse range of polyphenols with significant biological functions. Ferulic acid has been extensively studied for its ability to regulate lipid metabolism, exert antioxidant effects, and reduce inflammation [92]. *P*-coumaric acid and gallic acid, also contribute to various health benefits, for example, it has been studied because of its help reducing depression-like symptoms caused by long-term stress [156]. On the other hand, gallic acid, a well-known phenolic compound, exhibits strong antioxidant activity, along with antimicrobial and antitumor effects [157]. Additionally, other polyphenols, such as naringenin, demonstrate potent antioxidant activity and holds promise for pharmaceutical and nutraceutical applications due to its anti-inflammatory, antimicrobial, antidiabetic, anti-obesity, cardiovascular protective, neuroprotective, and anticancer properties [155]. Besides antioxidants, corn stover also works as an interesting source of plant-based protein, resulting in an alternative to animal-based sources for proteic foodstuff, in the next section, the protein contribution of these by-products will be explored.

### 3.4. Isolation of Proteins from Corn Stover

Proteins are complex polymers, made of 19 different α-amino acids and one imino acid, linked via amide bonds, also known as peptide bonds [158]. They play several roles in biological and food systems; for instance, proteins act as structural components of cells and organs, to transport nutrients in the body, to store amino acids, carbon, nitrogen, among other nutrients and roles [158]. Some corn stover components, such as corn silks, have been recognized as sources of zein, a hydrophobic prolamine [16,94]. Zein is a safe, non-toxic, biocompatible, and degradable protein with an unique amino acid composition that includes a high proportion of hydrophobic amino acids and a deficiency in charged acidic, basic, and polar residues, which contributes to its high solubility [12].

Corn silks have been studied as alternative sources of plant-based protein. For example, Singh and coworkers [17] determined the protein and ash content in corn silk powder (15.29 ± 1.23 wt.% protein and 5.29 ± 0.29 wt.% ash), with protein values within the range quantified in other studies about corn silk [82]. They also performed differential scanning calorimetry (DSC), by heating the powder from 30 °C to 450 °C; this confirmed that corn silk powder is highly stable in this range, with a glass transition temperature of T_g_ = 277.5 °C, which is desirable for protein-rich ingredients in food applications [17]. In another study, Egyptian corn silk fibers were used to extract zein protein and cellulose through two different protocols: filtration with NaOH/H_2_SO_4_ and solubilization with NaOH/Ure. Both methods proved effective for isolating these biomolecules, and the extracted zein protein exhibited promising antioxidant activity, likely due to their naturally high phenolic content [16]. The presence of these phenolic compounds may enhance the bioactive properties of the extracted protein, suggesting that corn silk proteins could provide both nutritional and functional benefits in food formulations [159].

Orellana-Palacios et al. [20] studied corn silk as a potential nutritional source. They found that the dried corn silk contained approximately 20.3 ± 1.7 wt.% of total protein, primarily composed of glutelins (67.5%), followed by albumins (23.3%) and globulins (9.23%). Thermal analysis indicated a relatively low denaturation temperature, suggesting suitability for food applications. However, the amino acid profile revealed deficiencies in essential amino acids recommended by FAO/WHO, particularly lysine. Overall, the study suggests that corn silk and its fractions may serve as valuable nutritional and functional ingredients in food and related products.

Although corn silk has been extensively studied as a source of bioactive proteins, research on protein extraction from other corn stover components, such as corn cobs, corn stalks, and corn leaves, remains scarce. These components are primarily composed of lignocellulosic material, making protein isolation more challenging compared to softer plant tissues. While some investigations have explored their potential as animal feed due to their residual protein content, efficient and scalable methods for extracting and purifying proteins from them have not been widely developed. As a result, further research is needed to assess the feasibility of utilizing these underexplored agricultural residues as protein sources.

In this sense, aligning the choice of feedstock with the target biomolecule ensures improved yield and cost-effectiveness [160], while diverting effort away from materials that lack sufficient compositional value [161]. Moreover, this selection should be guided by the intended application: while pharmaceutical or cosmetic industries may benefit from high purity extracts even at low yields, sectors, such as food and agriculture require bulk extraction from rich sources to ensure scalability and economic viability [161]. Table 5 summarizes the assessment done by the authors regarding the most promising applications associated with each corn stover component. 

## 4. Food Applications of Biomolecules from Corn Stover

Among the uses that have been given to corn stover, its application in food has been marked by the exploitation of its phytochemicals, reported in Table 1. In the work by Sari et al. [121], the authors made use of the film-forming properties found in corn cob starch, developing an edible film that used glycerol and sorbitol as plasticizers to develop edible films. The produced films showed practical qualities for packaging while addressing environmental concerns related to plastic waste [121]. In another work, Yan and coworkers [37] studied different ways to extract xylooligosaccharides from corn cob, corn leaves and corn stalk, and reported the prebiotic activity of XOS, highlighting its role as a carbon source for probiotics to promote multiple functional effects, such as optimizing intestinal flora, promoting intestinal health, reinforcement of the intestinal barrier, stimulation of immune responses, enhancement of daily weight gain in animals, improvement of feed conversion efficiency, and augmentation of antioxidant capacity [39,162,163].

Between these properties, the prebiotic capacity of XOS derived from corn stover has attracted the attention of several studies, especially due to their capacity to selectively stimulate beneficial gut microbiota [37]. For instance, XOS supplementation from corn cobs was shown to promote a higher growth rate of *Enterococcus faecium* [39]. In addition, when administered in combination with *Lactiplantibacillus paraplantarum* S2, XOS increased the abundance of live *Lactobacillus* and *Bifidobacterium* in mice feces, while reducing populations of *Enterococcus*, *Enterobacterium*, and *Clostridium* [37,164]. Moreover, corn cob XOS supported the proliferation of *Bifidobacterium adolescentis*, *B. bifidum*, *Lactobacillus fermentans*, and *L. acidophilus*, with a more pronounced effect observed in *Bifidobacterium* species [37]. Similarly, XOS obtained from corn stover were shown to act as a carbon source for *Bifidobacterium*, *Lactobacillus*, *Enterococcus*, and *Bacteroides prevotella*, further evidencing their prebiotic activity [162].

Irmak et al. [38] also evaluated xylose from corn stover for xylitol production, a pentose sugar alcohol used as a sugar substitute in the food industry because of a sweetening power close to sucrose; a caloric value of 2.4 kcal/g, compared to the 4 kcal/g of sucrose, and antimicrobial properties, linked to dental health benefits and anti-carcinogenic properties [165], these properties can be transferred to pharmaceutical applications. It is also used as an intermediate in the synthesis of a variety of commodity chemicals [38]. The extraction process of xylitol was achieved through hydrolysis of corn fractions for later reduction of the xylose in the biomass hydrolysate into xylitol using of NaBH_4_ as reagent. Xylitol content of the hydrolysates after reduction was higher in corn cob (0.14 mg/mL), followed by husk (0.10 mg/mL) and stover (0.08 mg/mL), however, raw materials as corn bran (0.79 mg/mL) gave better yields in the extraction process, further studies on xylitol production from corn stover are necessary to develop a competitive method to obtain xylitol.

After polysaccharides, polyphenols are the most widely used compounds derived from corn stover. Most of works aim to optimize the extraction process; for instance, Vijayalaxmi et al. [36] found that methanol (50% (*w*/*v*)) was the most suitable solvent for the extraction of polyphenols when compared to different concentrations of ethanol and water, yielding 6% extract, with a TPC of 48.50 g GAE/100 g extract, a total tannin content of 38.12 g TAE/100 g extract and a total flavonoid content of 8.23 g RE/100 g extract. 

Other studies like the one from Moreira et al. [69], investigated an active extract from corn cob to impart antimicrobial activity to a bioactive packaging. The extract showed bactericidal activity against *Escherichia coli* and *Staphylococcus aureus* at a concentration of 4% (*w*/*v*). After evaluating its antimicrobial activity, the extracts were incorporated into sodium alginate films, where several physicochemical and functional parameters evaluated. Film thickness, a critical factor influencing mechanical strength and gas transfer rates, was higher in films containing corn cob extract (185.08 μm) compared to the control films (121.5 μm). In the case of film solubility in water, both control and extract-containing films displayed solubility values of approximately 70% at 25 °C, indicating that the incorporation of antioxidant compounds did not significantly alter the water solubility profile of the alginate matrix. Finally, antimicrobial activity of the active films was confirmed through inhibition zone assays, where films containing the antioxidant extracts exhibited inhibition zones of 14.3 mm against *E. coli* and 14.9 mm against *Listeria monocytogenes*, while control films showed no inhibitory effect. These results indicated that the antimicrobial action originated exclusively from the bioactive compounds present in the corn cob extract, underscoring its potential as functional additives in the development of active packaging materials.

Besides the isolation and use of specific biomolecules, it is also important to recognize that corn stover flour also enhances the functional and health-promoting properties in food, due to their high dietary fiber content and antioxidant activity, making them attractive candidates for direct incorporation into human food formulations [71,166]. Paick et al. [167] studied the dietary fiber in corn stover for its incorporation into food matrices, by incorporating corn silk into a muffin formulation. Wheat flour was partially replaced with corn silk powder at levels of 10, 20, 30, and 40 wt.%. It was found that as the proportion of corn silk increased, the content of protein, crude fiber, and phenolic content in the muffins increased, with the highest phenolic concentration observed at a 40 wt.% substitution level. The authors performed a sensory evaluation to determine consumer acceptability, revealing that the addition of corn silk enhanced key attributes such as color, aroma, mouthfeel, texture, and overall appeal.

Shao [71] patented an invention using corn silk and husk powders to produce biscuits rich in dietary. The formulation included 50 parts broomcorn millet flour, 20 parts quinoa flour, 20 parts low-gluten wheat flour, 15 parts corn germ powder, 7 parts corn silk powder, 10 parts corn husk powder, and 5 parts ginkgo leaf powder as primary ingredients. Mice fed with the biscuits containing corn silk and corn husk powder showed a 15–20% reduction in body weight compared to control groups over the experimental period. The patent claims the biscuits aid in weight and fat loss due to their high fiber, low-calorie content, and prebiotic-rich ingredients, promoting satiety and reducing fat absorption [71].

Although different studies are increasingly focusing on the potential use of corn stover in the food industry, there are some challenges, commonly neglected in scientific literature, that should be assessed to incorporate corn stover in a circular economy for the food industry. The following section will discuss the challenges that should be addressed for the use of corn stover to transition from research to application.

## 5. Challenges and New Perspectives

**Figure 4:**
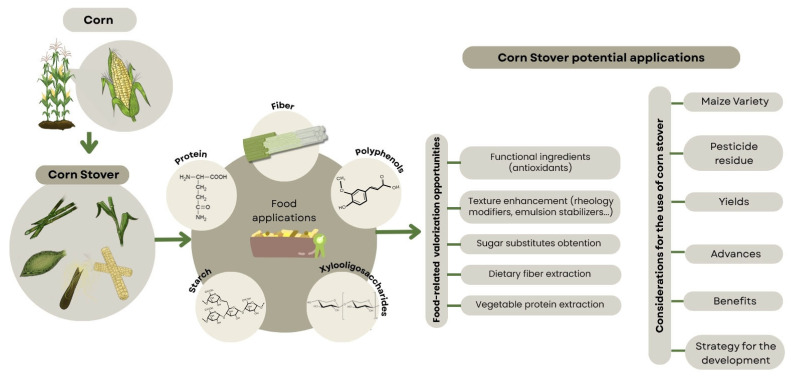
Schematic depiction of challenges to be solved in order to incorporate corn stover in circular economies.

Currently, the paths to exploit corn by-products focus on two alternatives. The first uses corn stover for biodiesel production or for developing biomaterials such as plastics and hydrogels [168]. The second is centered on the valorization of corn processing by-products, including corn gluten meal and corn steep liquor [12]. However, as mentioned before, both perspectives are limited and overlook a wide range of possibilities, particularly the potential that corn stover holds in other fields such as food production and agro-industrial applications, as demonstrated by the wide array of biomolecules present in corn stover and the challenges to be solved for their exploitation (Figure 4) [36,37,39,50,73]. Thus, the valorization of corn stover should be oriented toward the design of functional ingredients or dietary supplements, addressing nutritional challenges across both developing and developed regions [169]. Future research is expected to focus on extracting biomolecules from corn stalks and incorporating them into functional food matrices, including baked goods, beverages, and supplements [35,38,71], to enhance the functional properties of traditional foods while meeting consumer demand for health-promoting and natural additives.

Nonetheless, to strengthen the applications of corn stover in food and other sectors, knowledge gaps shall be properly addressed. For example, on a local level, the Corn Chain Productive Management Plan in Colombia does not explicitly incorporate corn stover as part of the productive chain, nor does it provide specific strategies for their management or valorization [170]. This reinforces the concept of corn stover as “waste” rather than as potential ingredients or raw materials, hindering their integration into agro-industrial or environmental applications. Addressing this lack of strategies for waste management in national plans is crucial to reposition corn stover as input with strategic value and to enable their systematic inclusion within the corn value chain.

Furthermore, one of the most critical gaps in the current literature relates to the potential presence of agrochemicals such as herbicides, fungicides, and pesticides, in corn stover [171], as exposure to these substances has been linked to an increase in human diseases, such as cancer, Alzheimer’s or Parkinson [172], caused by genetic and epigenetic changes through the modification of various processes at cellular level [173]. Cao et al. [174], studied samples of corn stover produced in China, finding that residues of atrazine (ATR) occasionally exceeded the national maximum residue limit (MRL ~0.01 mg/kg), with values reaching up to 0.17 mg/kg. 

Similar preoccupations apply to other contaminants, including heavy metals and mycotoxins, which may accumulate in corn stover due to their presence in the soil [175,176]. Besides, the presence of these residues in unprocessed corn stover is particularly critical, since processing operations such as dehydration may lead to an increased concentration of these components, and transformations of pesticide molecules can occur under the influence of temperature, pH, or microbial activity, potentially generating secondary contaminants with unknown toxicological profiles [13,177]. These findings justify the monitorization and further study of pesticide accumulation in agricultural residues [178] to ensure consumer safety when used for food applications [179]. 

Another aspect that is often overlooked is the influence of the maize variety in the properties and biomolecules present in the corn stover. Even though this crop is native to Mesoamerica [180], agricultural policies in recent decades have favored the widespread use of a few improved and hybrid races, leading to a reduction in biodiversity and a concern dependence on imported seeds [181]. The importance of identifying and exploring different varieties of corn is highlighted in purple varieties, which present higher levels of polyphenols and antioxidant compounds, making them more appropriate for the isolation of those compounds and the possibility of scaling-up these processes to an industrial capacity [140]. 

Academic and research institutions should prioritize programs that aim to strengthen these traditional varieties. For instance, Del Carmen-Bravo and colleagues [181] developed composite varieties from outstanding native populations of corn, intending to improve their agronomic performance as well as enable these varieties to compete with commercial cultivars [182]. Additionally, the value of native maize varieties extends to their unique sensory attributes, such as flavor, color, and texture, that are essential in the traditional cuisine of various regions and enable the preparation of differentiated and culturally significant foods.

Alongside these knowledge gaps, scalability remains a key point. Most studies to date are limited to laboratory-scale experiments, often using conditions that are not easily transferable to industrial settings, for instance, high energy inputs, non-scalable chemical and physical treatments [183]. The lack of standardized extraction protocols, including wide variations in solvents, pH, temperature, pretreatments and processing times, make cross-study comparison difficult and hinder the development of transferable technologies, as explored by authors such as Caruso et al. [184] in industries like fishery. Scaling these methods requires process optimization, development of technologies with balance between economic and industrial viability, and integration into a circular biorefinery framework, where multiple streams can be valorized simultaneously to reduce costs [185].

Biorefinery processes should consider isolating various biomolecules rather than targeting a single constituent to enable full material exploitation and improve biomolecule extraction yields. They also would benefit from using multiple sources of agro-industrial by-products as the profitability of bioprocesses is typically achieved at high production capacities [186]. An example is provided by Gullón et al. [187], who employed by-products and wastewater from the olive industry. They first isolated antioxidants from solid olive residues, then, autohydrolysis converted the remaining biomass into a liquor rich in oligosaccharides and a solid fraction containing cellulose and lignin. From the liquid phase and the olive-processing wastewater, the authors successfully obtained purified oligosaccharides and antioxidants. 

Social awareness also plays a critical role in the successful transition from the laboratory to industrial deployment, given that one persistent challenge associated with corn stover is the collection of its components, as farmers commonly leave the by-products dispersed across the fields without designated storage or aggregation points. This practice creates logistical barriers, limiting a consistent supply of feedstock and constraining the scalability of agro-industrial by-product valorization [186]. In this context, government policies and collaborations among sectors and stakeholders are crucial since alignment across institutional and societal visions can facilitate the scalability of these processes [188]. Moreover, clear communication of the environmental, economic, and technological benefits associated with valorizing agro-industrial by-products is essential to foster community acceptance and encourage market adoption.

## 6. Market Perspectives

These challenges are not easy to overcome, but achieving them could expand the opportunities to valorize corn stover and provide additional and valuable income alternatives for farmers. For example, the market for functional ingredients, which is especially important for the biomolecules discussed here, was valued at USD 124.23 billion in 2023 and is expected to reach USD 177.37 billion by 2028 [189]. Inside this market, the segment of insoluble dietary fiber expanded in the international market between 2013 and 2023, where imports in Colombia increased by approximately USD 6.7 million in trade value, reflecting a growing reliance on this additive [190]. Thus, the valorization of corn stover offers a cost-effective pathway for meeting this demand while simultaneously strengthening the agro-industrial value chain [190]. 

**Figure 5:**
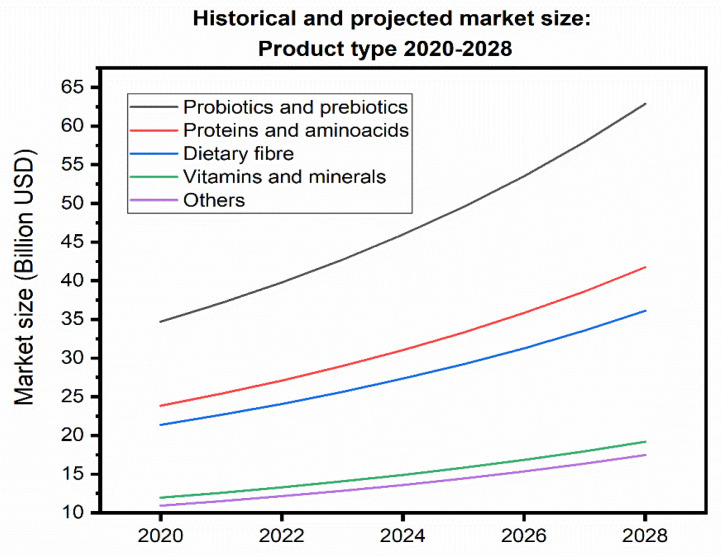
Historical size and market projection: Type of product in the years 2020–2028 (the figures are given in billions of USD) [189].

Beyond insoluble dietary fiber, other segments of interest in the functional ingredients market as probiotics and prebiotics, and antioxidants stand to benefit from the incorporation of lignocellulosic fibers, xylooligosaccharides, and polyphenols derived from husks, cobs, and silks, respectively. The market size for each of these segments, along with their projected growth by 2028, is shown in Figure 5. 

This behavior offers cost effective solutions that adhere to the three pillars of sustainability [169]. In the social aspect, food by-products serve as an affordable and available source of bioactive compounds with health benefits; in the economic component, this approach could improve the financial conditions of farmers and producers by adding value to food waste, reducing the cost of formulated products, and minimizing reliance on synthetic chemicals. Finally, the environmental impact of these agricultural applications is reduced by using their by-products, as there is a reduction in the inadequate disposal of organic matter.

It is worth noting that the utilization of agro-industrial by-products in food is not an unexplored field but rather a well-established reality in the food industry. Many fruits, vegetables, and cereals have been pioneers in this area due to their high content of bioactive compounds and nutritional value. A clear example is cocoa husk, which is rich in polyphenols, dietary fiber, and other bioactive compounds. This has driven the development of studies and patents focused on its use as a functional ingredient in enriched foods and dietary supplements [191]. Similarly, by-products from fruits such as grapes, berries, kiwi, and melon, as well as those from cereals, such as rice and certain vegetables, have been incorporated into the formulation of fortified bakery products, dairy, and meat products [169]. Thus, the use of corn stover and its related biomolecules in food applications will strengthen the use of by-products in the context of sustainability.

## 7. Conclusions

This review highlighted the compositional variety and valorization potential of corn stover. Although these materials are frequently relegated to low-value uses such as landfilling and biomass burning, they contain a diverse array of biomolecules, including lignocellulosic fibers, non-cellulosic polysaccharides, proteins, and polyphenols, with considerable potential for value-added applications across multiple sectors. The distribution of these biomolecules varies markedly among different anatomical parts of the plant; for instance, stalks and cobs are primarily composed of structural carbohydrates, while corn silks exhibit higher concentrations of proteins and polyphenolic compounds. This inherent biochemical heterogeneity highlights the need for thorough characterization and fractionation of corn stover, enabling the design of targeted and efficient extraction strategies.

In food applications, these biomolecules contribute to the functional properties of the resultant product. Lignocellulosic fibers serve as dietary fiber sources. Non-cellulosic polysaccharides, such as xylooligosaccharides, provide low-calorie sweetness and prebiotic benefits. Polyphenols act as natural antioxidants, while proteins contribute to both nutritional value and texture enhancement. Nevertheless, a significant barrier to their incorporation in food systems is the potential presence of pesticide residues, including commonly used compounds such as organophosphates and pyrethroids. While the study of the interaction biomolecule-matrix and scale-up of the developed formulations significantly hinders the adoption of these components as a food ingredient. These developments are important to achieve efficient use of the crop, aiding in tackling the future threats to food security.

## Figures and Tables

**Table 3:** Lignocellulosic fiber composition in corn stover.

Corn by-Product	Cellulose	Hemicellulose	Lignin	Reference
	(wt.%) ^1^			
**Corn husk**	~29–38	~40–45	~7–12	[12,18]
**Corn leaves**	~19	~40	~13	[19]
**Corn stalk**	~26–28	~34–36	~16–20	[19,84]
**Corn cob**	~18–30	~33–45	~16–22	[19,85,101]

^1^ Values were rounded up to the nearest full percents.

**Table 5:** Promising food applications by corn stover part.

Corn Stover Component	Promising Food Application	Responsible Biomolecule	Reference
Silk	Antioxidants	Polyphenols	[36,50,73,74]
	Vegetable protein source	Protein	[17,20]
	Dietary fiber for bakery products	Structural and non-structural biopolymers	[71]
Cob	Sugar substitutes	Xylooligosaccharides	[37]
	Texture enhancement in food products	Starch	[118]
	Edible films production		[121]
Husk	Dietary fiber	Structural and non-structural biopolymers	[129]
	Rheology modifier	Cellulose	[113]
	Emulsion stabilizer		[109]
Leaves and stalks	Sugar substitutes	Xylooligosaccharides	[37]
	Biodegradable materials for food packaging	Lignocellulosic fiber	[72]

## Data Availability

No new data were created or analyzed in this study. Data sharing is not applicable to this article.
